# 4-Methyl-*N*′-(2,2,2-trichloro­ethanimido­yl)benzene-1-carboximidamide

**DOI:** 10.1107/S1600536811044710

**Published:** 2011-11-02

**Authors:** Tracey L. Roemmele, René T. Boeré

**Affiliations:** a Department of Chemistry and Biochemistry, University of Lethbridge, Lethbridge, AB, Canada T1K 3M4

## Abstract

The title compound, C_10_H_10_Cl_3_N_3_, features a delocalized unsaturated N C N C N chain and strong intra­molecular N—H⋯N hydrogen bonding across the chelate ring and also intra­molecular N—H⋯Cl contacts to a CCl_3_-group Cl atom. The only inter­molecular contacts in the lattice are non-classical hydrogen bonds between methyl and CCl_3_ groups. The pseudo-six-membered ring is distinctly non-planar by virtue of rotation about the N—C bond between the carboximidamide and imine components [C—N—C—N torsion angle = −23.6 (2) °].

## Related literature

For crystal structures of closely related *N*′-(trichloro/tri­fluoro­ethanimido­yl)aryl-1-carboximidamides, see: Boeré, Roemmele, Suduweli Kondage *et al.* (2011) ▶; Boeré, Roemmele & Yu (2011 ▶). For a review of this less-common class of chelating ligands, see: Kopylovich & Pombeiro (2011 ▶).
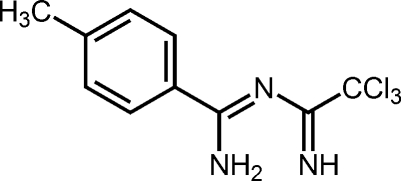


## Experimental

### 

#### Crystal data

C_10_H_10_Cl_3_N_3_
*M* *_r_* = 278.56
Monoclinic, 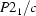

*a* = 12.2952 (7) Å
*b* = 9.0696 (6) Å
*c* = 11.5407 (7) Å
β = 109.661 (1)°
*V* = 1211.90 (13) Å^3^
*Z* = 4
Mo *K*α radiation
μ = 0.73 mm^−1^
*T* = 173 K
0.43 × 0.34 × 0.25 mm

#### Data collection

Bruker APEXII CCD area-detector diffractometer
Absorption correction: multi-scan (*SADABS*; Bruker, 2008 ▶) *T*_min_ = 0.747, *T*_max_ = 0.838
16754 measured reflections
2762 independent reflections
2594 reflections with *I* > 2σ(*I*)
*R*_int_ = 0.014

#### Refinement

*R*[*F* ^2^ > 2σ(*F* ^2^)] = 0.029
*wR*(*F* ^2^) = 0.077
*S* = 1.08
2762 reflections
158 parameters
H atoms treated by a mixture of independent and constrained refinement
Δρ_max_ = 0.40 e Å^−3^
Δρ_min_ = −0.41 e Å^−3^

### 

Data collection: *APEX2* (Bruker, 2008 ▶); cell refinement: *SAINT-Plus* (Bruker, 2008 ▶); data reduction: *SAINT-Plus*; program(s) used to solve structure: *SHELXS97* (Sheldrick, 2008 ▶); program(s) used to refine structure: *SHELXL97* (Sheldrick, 2008 ▶); molecular graphics: *Mercury* (Macrae *et al.*, 2006 ▶); software used to prepare material for publication: *publCIF* (Westrip, 2010 ▶).

## Supplementary Material

Crystal structure: contains datablock(s) I, global. DOI: 10.1107/S1600536811044710/pv2466sup1.cif

Structure factors: contains datablock(s) I. DOI: 10.1107/S1600536811044710/pv2466Isup2.hkl

Additional supplementary materials:  crystallographic information; 3D view; checkCIF report

## Figures and Tables

**Table 1:** Hydrogen-bond geometry (Å, °)

*D*—H⋯*A*	*D*—H	H⋯*A*	*D*⋯*A*	*D*—H⋯*A*
N1—H2⋯N3	0.83 (2)	2.07 (2)	2.683 (2)	130.8 (18)
N3—H3⋯Cl1	0.81 (2)	2.49 (2)	2.9993 (15)	122.2 (19)
C10—H10*A*⋯Cl2^i^	0.98	2.93	3.871 (2)	162 (1)
C10—H10*C*⋯Cl3^ii^	0.98	2.93	3.581 (2)	125 (1)

Symmetry codes: (i) 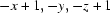; (ii) 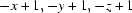.

**Fig. 1.**
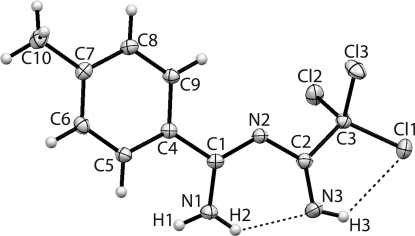
Displacement ellipsoid plot (drawn at the 30% probability level) of the title compound. Intramolecular N1···N3 and N3···Cl1 hydrogen bonds (dotted lines) occur in the molecular structure within the crystal lattice.

**Fig. 2.**
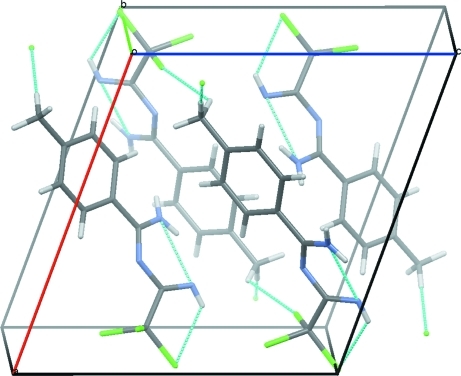
Crystal packing diagram showing the short intermolecular contacts as well as the intramolecular hydrogen bonds.

## supplementary crystallographic information

### Comment

The title compound (Figure 1), commonly known as an imidoylamidine, was prepared as part of our continuing interest in *C*,*N*,*S* based heterocycles (Boeré, Roemmele & Yu, 2011). The molecular structure is very similar to seven independent molecules from our prevous study (Boeré, Roemmele, Suduweli Kondage *et al.*, 2011), with only a single tautomeric form being evidenced in the crystal lattice. The bond lengths and angles for the N≐ C≐N≐C≐N core are nearly identical within the s.u. values to the comparison group. Intramolecular hydrogen bonding occurs between N1—H2···N3 at 2.683 (2) Å to form a *pseudo* six membered ring. This hydrogen bond is slightly longer (just outside the s.u. values) than the previously reported average of 2.638 (14) Å, which is likely due to a slight rotation of the core (C1—N2—C2—N3 torsion angle = 23.6 (2) °) which causes N3 to be twisted out of the plane of the other four atoms (N1,C1,N2,C2) compared to a range of 4.2 (5) to 16.0 (2) ° for this torsion angle in the comparison group. Also noteworthy is a short intramolecular N3—H3···Cl1 contact at 2.9993 (15) Å, which is within s.u. values of the other known trichloromethyl imidoylamidines. The most surprising feature is the complete lack of intermolecular hydrogen bonding involving the NH or NH_2_ groups, which was previously observed only in the structure of 4-trifluormethyl-*N*'-(2,2,2-trichloroethanimidoyl)benzene-1-carboximidamide. In fact, the only short contacts are non-classical hydrogen bonds between the methyl and CCl_3_ groups, specifically from H10A to Cl2^i^ and H10C to Cl3^ii^ at 2.9272 (5) and 2.9245 (5) Å (Figure 2).

### Experimental

The title compound was prepared as were those described in Boeré, Roemmele, Suduweli Kondage *et al.* (2011) by addition of trichloroacetonitrile to *N,N,N*'-4-methylbenzamidine in acetonitrile. Crystals suitable for X-ray diffraction were grown by vacuum sublimation (m.p. 315–317 K).

### Refinement

C-bound H atoms were treated as riding, with C–H = 0.98 Å and *U*_iso_(H) = 1.5*U*_eq_(C) for methyl and C–H = 0.95–0.96 Å and *U*_iso_(H) = 1.2*U*_eq_(C) for all other H atoms. The three N-bound H-atom positions were refined using a distance restraint of 0.88 Å and with *U*_iso_(H) = 1.2*U*_eq_(N).

### Crystal data

**Table:**

C_10_H_10_Cl_3_N_3_	*F*(000) = 568
*M**_r_* = 278.56	*D*_x_ = 1.527 Mg m^−^^3^
Monoclinic, *P*2_1_/*c*	Melting point: 315 K
Hall symbol: -P 2ybc	Mo *K*α radiation, λ = 0.71073 Å
*a* = 12.2952 (7) Å	Cell parameters from 9876 reflections
*b* = 9.0696 (6) Å	θ = 2.9–27.5°
*c* = 11.5407 (7) Å	µ = 0.73 mm^−^^1^
β = 109.661 (1)°	*T* = 173 K
*V* = 1211.90 (13) Å^3^	Prism, colourless
*Z* = 4	0.43 × 0.34 × 0.25 mm

### Data collection

**Table:**

Bruker APEXII CCD area-detector diffractometer	2762 independent reflections
Radiation source: X-ray, Bruker D8	2594 reflections with *I* > 2σ(*I*)
graphite	*R*_int_ = 0.014
Detector resolution: 0.015 pixels mm^-1^	θ_max_ = 27.5°, θ_min_ = 1.8°
phi and ω scans	*h* = −15→15
Absorption correction: multi-scan (*SADABS*; Bruker, 2008)	*k* = −11→11
*T*_min_ = 0.747, *T*_max_ = 0.838	*l* = −14→14
16754 measured reflections	

### Refinement

**Table:**

Refinement on *F*^2^	Primary atom site location: structure-invariant direct methods
Least-squares matrix: full	Secondary atom site location: difference Fourier map
*R*[*F*^2^ > 2σ(*F*^2^)] = 0.029	Hydrogen site location: inferred from neighbouring sites
*wR*(*F*^2^) = 0.077	H atoms treated by a mixture of independent and constrained refinement
*S* = 1.08	*w* = 1/[σ^2^(*F*_o_^2^) + (0.0354*P*)^2^ + 0.6197*P*] where *P* = (*F*_o_^2^ + 2*F*_c_^2^)/3
2762 reflections	(Δ/σ)_max_ = 0.001
158 parameters	Δρ_max_ = 0.40 e Å^−^^3^
0 restraints	Δρ_min_ = −0.41 e Å^−^^3^

### Special details

**Table:**

Experimental. A crystal coated in Paratone oil was mounted on the end of a thin glass capillary and cooled in the gas stream of the diffractometer Kryoflex device.
Geometry. All e.s.d.'s (except the e.s.d. in the dihedral angle between two l.s. planes) are estimated using the full covariance matrix. The cell e.s.d.'s are taken into account individually in the estimation of e.s.d.'s in distances, angles and torsion angles; correlations between e.s.d.'s in cell parameters are only used when they are defined by crystal symmetry. An approximate (isotropic) treatment of cell e.s.d.'s is used for estimating e.s.d.'s involving l.s. planes.
Refinement. Refinement of *F*^2^ against ALL reflections. The weighted *R*-factor *wR* and goodness of fit *S* are based on *F*^2^, conventional *R*-factors *R* are based on *F*, with *F* set to zero for negative *F*^2^. The threshold expression of *F*^2^ > σ(*F*^2^) is used only for calculating *R*-factors(gt) *etc*. and is not relevant to the choice of reflections for refinement. *R*-factors based on *F*^2^ are statistically about twice as large as those based on *F*, and *R*- factors based on ALL data will be even larger.

### Fractional atomic coordinates and isotropic or equivalent isotropic displacement parameters (Å^2^)

**Table:**

	*x*	*y*	*z*	*U*_iso_*/*U*_eq_	
C1	0.60366 (11)	0.08801 (14)	0.75586 (12)	0.0251 (3)	
C2	0.78479 (12)	0.15175 (16)	0.89254 (13)	0.0296 (3)	
C3	0.89517 (12)	0.22286 (16)	0.88006 (12)	0.0282 (3)	
C4	0.50862 (11)	0.12222 (14)	0.63920 (12)	0.0247 (3)	
C5	0.39803 (12)	0.06308 (16)	0.61262 (13)	0.0308 (3)	
H5	0.3829	−0.0045	0.6682	0.037*	
C6	0.31014 (12)	0.10232 (17)	0.50565 (14)	0.0330 (3)	
H6	0.2354	0.0609	0.4888	0.040*	
C7	0.32937 (12)	0.20122 (16)	0.42266 (13)	0.0299 (3)	
C8	0.44024 (12)	0.25899 (17)	0.44926 (13)	0.0313 (3)	
H8	0.4552	0.3263	0.3934	0.038*	
C9	0.52899 (12)	0.22015 (16)	0.55551 (13)	0.0283 (3)	
H9	0.6040	0.2603	0.5715	0.034*	
C10	0.23320 (14)	0.2455 (2)	0.30785 (15)	0.0413 (4)	
H10A	0.1764	0.1656	0.2828	0.062*	
H10B	0.2648	0.2651	0.2419	0.062*	
H10C	0.1957	0.3347	0.3240	0.062*	
N1	0.59080 (12)	−0.02814 (15)	0.82081 (13)	0.0339 (3)	
N2	0.69301 (10)	0.17656 (14)	0.78685 (11)	0.0298 (3)	
N3	0.78334 (13)	0.08069 (19)	0.98683 (13)	0.0434 (3)	
Cl1	1.01604 (3)	0.20856 (5)	1.01601 (3)	0.04137 (12)	
Cl2	0.92765 (3)	0.12843 (5)	0.76109 (4)	0.04211 (12)	
Cl3	0.87281 (4)	0.41134 (4)	0.84076 (5)	0.04654 (12)	
H1	0.5360 (18)	−0.087 (2)	0.7925 (18)	0.045 (5)*	
H2	0.6389 (17)	−0.041 (2)	0.8902 (18)	0.039 (5)*	
H3	0.846 (2)	0.083 (3)	1.041 (2)	0.055 (6)*	

### Atomic displacement parameters (Å^2^)

**Table:**

	*U*^11^	*U*^22^	*U*^33^	*U*^12^	*U*^13^	*U*^23^
C1	0.0274 (6)	0.0241 (6)	0.0277 (6)	0.0044 (5)	0.0141 (5)	0.0011 (5)
C2	0.0292 (7)	0.0314 (7)	0.0289 (7)	−0.0001 (5)	0.0107 (5)	0.0021 (5)
C3	0.0278 (6)	0.0279 (7)	0.0279 (6)	0.0003 (5)	0.0080 (5)	0.0000 (5)
C4	0.0263 (6)	0.0229 (6)	0.0271 (6)	0.0036 (5)	0.0118 (5)	−0.0007 (5)
C5	0.0304 (7)	0.0297 (7)	0.0338 (7)	−0.0013 (5)	0.0130 (6)	0.0035 (6)
C6	0.0259 (6)	0.0352 (7)	0.0378 (8)	−0.0007 (6)	0.0105 (6)	−0.0002 (6)
C7	0.0291 (7)	0.0315 (7)	0.0292 (6)	0.0074 (5)	0.0100 (5)	−0.0009 (5)
C8	0.0332 (7)	0.0330 (7)	0.0300 (7)	0.0036 (6)	0.0137 (6)	0.0054 (6)
C9	0.0266 (6)	0.0300 (7)	0.0309 (7)	0.0014 (5)	0.0131 (5)	0.0015 (5)
C10	0.0324 (8)	0.0489 (9)	0.0384 (8)	0.0086 (7)	0.0066 (6)	0.0064 (7)
N1	0.0350 (7)	0.0292 (6)	0.0353 (7)	−0.0021 (5)	0.0090 (5)	0.0079 (5)
N2	0.0278 (6)	0.0333 (6)	0.0281 (6)	−0.0013 (5)	0.0091 (5)	0.0061 (5)
N3	0.0373 (7)	0.0599 (9)	0.0306 (6)	−0.0050 (7)	0.0080 (6)	0.0124 (6)
Cl1	0.0336 (2)	0.0485 (2)	0.0339 (2)	−0.00458 (15)	0.00062 (15)	−0.00111 (15)
Cl2	0.0371 (2)	0.0550 (3)	0.0387 (2)	−0.00594 (17)	0.01858 (16)	−0.01338 (17)
Cl3	0.0414 (2)	0.02872 (19)	0.0694 (3)	0.00018 (15)	0.01851 (19)	0.00905 (17)

### Geometric parameters (Å, °)

**Table:**

C1—N2	1.3098 (18)	C6—C7	1.389 (2)
C1—N1	1.3328 (18)	C6—H6	0.9500
C1—C4	1.4885 (18)	C7—C8	1.395 (2)
C2—N3	1.270 (2)	C7—C10	1.504 (2)
C2—N2	1.3732 (18)	C8—C9	1.3849 (19)
C2—C3	1.5525 (19)	C8—H8	0.9500
C3—Cl1	1.7657 (14)	C9—H9	0.9500
C3—Cl3	1.7662 (15)	C10—H10A	0.9800
C3—Cl2	1.7739 (14)	C10—H10B	0.9800
C4—C9	1.3955 (19)	C10—H10C	0.9800
C4—C5	1.3964 (19)	N1—H1	0.84 (2)
C5—C6	1.386 (2)	N1—H2	0.83 (2)
C5—H5	0.9500	N3—H3	0.81 (2)
			
N2—C1—N1	125.34 (13)	C6—C7—C8	118.11 (13)
N2—C1—C4	116.76 (12)	C6—C7—C10	120.94 (14)
N1—C1—C4	117.90 (13)	C8—C7—C10	120.94 (14)
N3—C2—N2	126.87 (14)	C9—C8—C7	121.33 (13)
N3—C2—C3	123.71 (13)	C9—C8—H8	119.3
N2—C2—C3	109.41 (11)	C7—C8—H8	119.3
C2—C3—Cl1	112.81 (10)	C8—C9—C4	120.22 (13)
C2—C3—Cl3	111.13 (10)	C8—C9—H9	119.9
Cl1—C3—Cl3	108.12 (8)	C4—C9—H9	119.9
C2—C3—Cl2	107.62 (10)	C7—C10—H10A	109.5
Cl1—C3—Cl2	108.12 (8)	C7—C10—H10B	109.5
Cl3—C3—Cl2	108.94 (8)	H10A—C10—H10B	109.5
C9—C4—C5	118.73 (13)	C7—C10—H10C	109.5
C9—C4—C1	119.31 (12)	H10A—C10—H10C	109.5
C5—C4—C1	121.92 (12)	H10B—C10—H10C	109.5
C6—C5—C4	120.46 (13)	C1—N1—H1	121.3 (14)
C6—C5—H5	119.8	C1—N1—H2	118.1 (14)
C4—C5—H5	119.8	H1—N1—H2	121 (2)
C5—C6—C7	121.15 (13)	C1—N2—C2	120.34 (12)
C5—C6—H6	119.4	C2—N3—H3	111.7 (16)
C7—C6—H6	119.4		
			
N3—C2—C3—Cl1	−5.2 (2)	C4—C5—C6—C7	−0.2 (2)
N2—C2—C3—Cl1	175.64 (10)	C5—C6—C7—C8	0.7 (2)
N3—C2—C3—Cl3	−126.84 (15)	C5—C6—C7—C10	−178.90 (14)
N2—C2—C3—Cl3	54.02 (14)	C6—C7—C8—C9	−0.4 (2)
N3—C2—C3—Cl2	113.95 (16)	C10—C7—C8—C9	179.23 (14)
N2—C2—C3—Cl2	−65.19 (13)	C7—C8—C9—C4	−0.5 (2)
N2—C1—C4—C9	15.26 (18)	C5—C4—C9—C8	1.0 (2)
N1—C1—C4—C9	−165.53 (13)	C1—C4—C9—C8	−176.86 (12)
N2—C1—C4—C5	−162.49 (13)	N1—C1—N2—C2	1.5 (2)
N1—C1—C4—C5	16.73 (19)	C4—C1—N2—C2	−179.38 (12)
C9—C4—C5—C6	−0.6 (2)	N3—C2—N2—C1	−23.6 (2)
C1—C4—C5—C6	177.12 (13)	C3—C2—N2—C1	155.50 (12)

### Hydrogen-bond geometry (Å, °)

**Table:**

*D*—H···*A*	*D*—H	H···*A*	*D*···*A*	*D*—H···*A*
N1—H2···N3	0.83 (2)	2.07 (2)	2.683 (2)	130.8 (18)
N3—H3···Cl1	0.81 (2)	2.49 (2)	2.9993 (15)	122.2 (19)
C10—H10A···Cl2^i^	0.98	2.93	3.871 (2)	162.(1)
C10—H10C···Cl3^ii^	0.98	2.93	3.581 (2)	125.(1)

Symmetry codes: (i) −*x*+1, −*y*, −*z*+1; (ii) −*x*+1, −*y*+1, −*z*+1.
